# Telomere Dynamics in Human Cells Reprogrammed to Pluripotency

**DOI:** 10.1371/journal.pone.0008124

**Published:** 2009-12-02

**Authors:** Steven T. Suhr, Eun Ah Chang, Ramon M. Rodriguez, Kai Wang, Pablo J. Ross, Zeki Beyhan, Shashanka Murthy, Jose B. Cibelli

**Affiliations:** 1 Cellular Reprogramming Laboratory, Department of Animal Science, Michigan State University, East Lansing, Michigan, United States of America; 2 Programa Andaluz de Terapia Celular y Medicina Regenerativa, Andalucía, Spain; University of Southern California, United States of America

## Abstract

**Background:**

Human induced pluripotent stem cells (IPSCs) have enormous potential in the development of cellular models of human disease and represent a potential source of autologous cells and tissues for therapeutic use. A question remains as to the biological age of IPSCs, in particular when isolated from older subjects. Studies of cloned animals indicate that somatic cells reprogrammed to pluripotency variably display telomere elongation, a common indicator of cell “rejuvenation.”

**Methodology/Principal Findings:**

We examined telomere lengths in human skin fibroblasts isolated from younger and older subjects, fibroblasts converted to IPSCs, and IPSCs redifferentiated through teratoma formation and explant culture. In IPSCs analyzed at passage five (P5), telomeres were significantly elongated in 6/7 lines by >40% and approximated telomere lengths in human embryonic stem cells (hESCs). In cell lines derived from three IPSC-teratoma explants cultured to P5, two displayed telomeres shortened to lengths similar to input fibroblasts while the third line retained elongated telomeres.

**Conclusions/Significance:**

While these results reveal some heterogeneity in the reprogramming process with respect to telomere length, human somatic cells reprogrammed to pluripotency generally displayed elongated telomeres that suggest that they will not age prematurely when isolated from subjects of essentially any age.

## Introduction

Somatic animal cells can be reprogrammed to pluripotency by nuclear transfer or introduction of reprogramming factors. These cells not only gain the capacity for differentiation into the multiple cell and tissue types that ultimately give rise to a complete embryo, but they also gain the capacity for essentially indefinite “rejuvenation”, or self-renewal. Telomeres are special structures at the ends of chromosomes that contain long tandem repeats of the DNA sequence TTAGGG and that reduce genetic instability with the passage of time (reviewed in [1]–[3]). Telomere length has emerged a critical indicator of replicative capacity and advancement of the aging process at both the cellular and organismal levels [4], [5], and elongation of telomeres has been reported in animal cells reprogrammed to pluripotency by both nuclear transfer [6]–[8] and direct reprogramming [9]. While reports such as these indicate that telomere elongation is a common feature of nuclear reprogramming, there are also reported exceptions. One such exception was Dolly the sheep that did not reset telomeres and displayed indicators of premature aging, revealing that in at least some cases, reprogramming can occur in the absence of telomere lengthening [10].

Human embryonic stem cells (hESCs), like their animal counterparts, also display elongated telomeres compared to differentiated somatic cells [11], [12]. Although there are relatively few studies of telomere lengths in human ESCs, hESC lines at the earliest passage reported – P15 – displayed average terminal restriction fragment (TRF) lengths of approximately 14 Kb that gradually declined and tended to level off at around 10 Kb between passages 40–80 [11], [13]. It has yet to be determined if human somatic cells directly reprogrammed to pluripotency respond similarly and display telomere lengths similar to hESCs. Directly reprogrammed human cells known as “induced pluripotent stem cells” (IPSCs) [14]–[16] may display no telomere elongation or shortened telomeres, elongation in some lines but not others, or significantly elongated telomeres equal to or exceeding those characteristic of hESCs. Before reprogrammed human somatic cells can be used to best advantage as models of disease *in vitro* or for therapeutic purposes, telomere dynamics need to be examined for human IPSCs.

**Figure 1:**
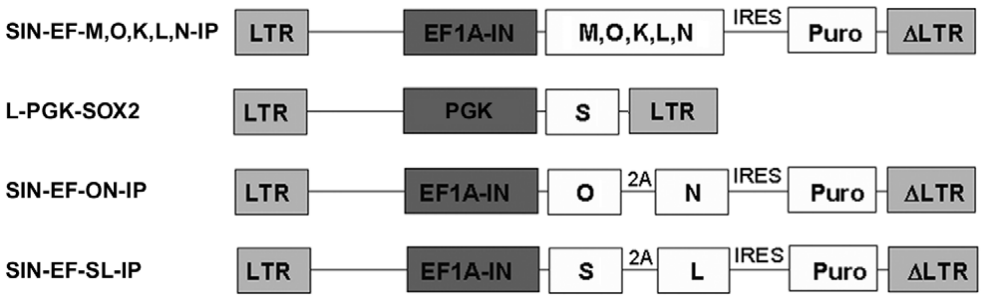
Lentiviral vectors used in IPSC production. LTR: viral LTRS, EF1A-IN: elongation factor 1-alpha promoter, PGK: PGK promoter, M: cMyc, O: Oct4, S: Sox2, K: KLF4, L: Lin28, N: Nanog transgenes. 2A: translation interruption element, IRES: internal ribosomal entry site, Puro: puromycin resistance gene.

IPSCs, like ESCs, have been shown in several reports to display increased activity of at least one important enzymatic component of telomere homeostasis – the reverse transcriptase telomerase (TERT) – compared to the activity seen in somatic cell types (i.e. [14], [15]). More recently it was shown that mouse fibroblasts reprogrammed to pluripotency have both TERT activity and elongated telomeres [9]. This group further demonstrated that although one component of the reprogramming cocktail, the oncogene c-myc, had been shown to directly activate telomerase expression in human cells [17], [18], it was not required for telomere elongation in mouse IPSCs. Marion and colleagues further demonstrated that fibroblasts from both young (6 month) and old donor mice (2.3 yr) elongate telomeres to a similar degree following IPSC conversion. Of note in this report is that although mouse IPSCs at low passage (i.e.<P8) displayed multiple indicators of reprogramming to pluripotency (including mESC-like morphology, alkaline phosphatase staining, and expression of mESC markers), they displayed only a small degree of telomere elongation relative to input fibroblasts. From P8, however, mIPSC telomeres progressively elongated until they had attained the lengths of mESC telomeres at about P30 . It was also noted that the inclusion of c-myc in the reprogramming mix accelerated telomere lengthening at early passages, but did not impact the overall length at later passage (Ref. [9], Figure 1I). Together, these results led the authors to justifiably conclude that “most telomere elongation occurs postreprogramming”[9].

We examined telomere length in human skin fibroblasts from young and old donor subjects, IPSCs derived from these cells, and IPSCs returned to a differentiated phenotype. We found that like animal cells reprogrammed by either somatic cell nuclear transfer or direct reprogramming, human fibroblasts converted to the IPSC phenotype generally displayed significantly elongated telomeres, and after re-differentiation, displayed a loss of telomere length. Like the mouse, this process was observed irrespective of the inclusion of c-myc in the reprogramming cocktail, and occured to approximately the same degree in cells derived from either young and old subjects. Unlike the mouse however, we observed greater heterogeneity between cell lines, both in the magnitude of telomere elongation during IPSC conversion and telomere shortening following redifferentiation. Also unlike the slow and progressive telomere elongation reported in mouse IPSCs, based on the seven human IPSC lines we analyzed, hIPSC telomeres achieved the 14–15 Kb length characteristic of human ESCs as early as P5.

## Results

### Input Fibroblasts, IPSCs, and Teratoma-Derived Cells Display Indicators of Their Respective Phenotypes

**Figure 2:**
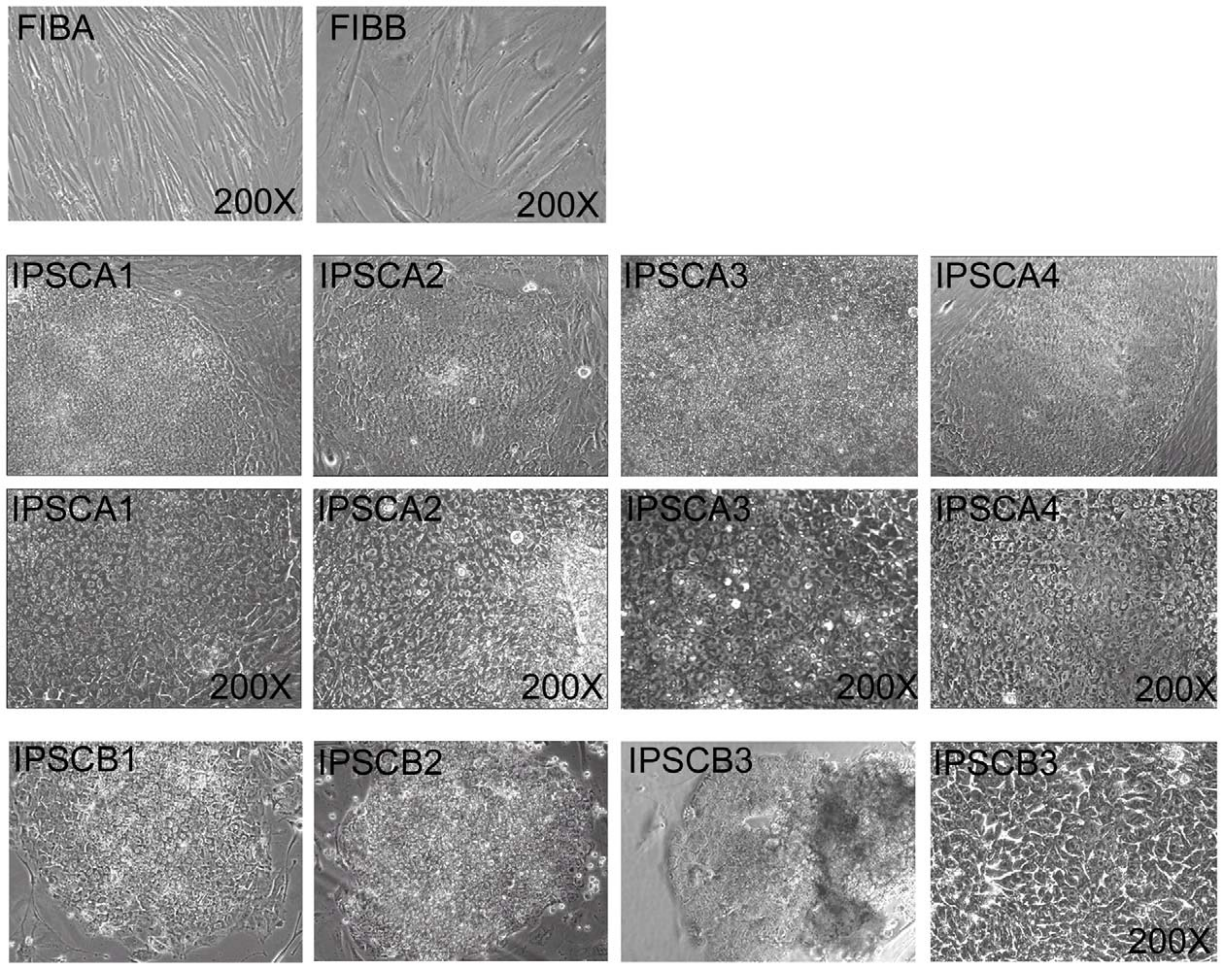
Phase-contrast images of FIB and IPSC lines used in this report (as labeled). FIBA and FIBB displayed a flat stellate cytoplasm with irregular edges characteristic of fibroblast cell types (200X). IPSC lines displayed round colonies with regular edges evident in low magnification 40X images that were composed of tightly packed cells with prominent nucleoli (200X, lower panels of IPSC lines) that appeared morphologically homogenous within the center of the colony and flattened toward the edges where they were bounded by the MEF feeder layer. IPSCB1 and IPSB2 are shown only at 40X magnification.

**Figure 3:**
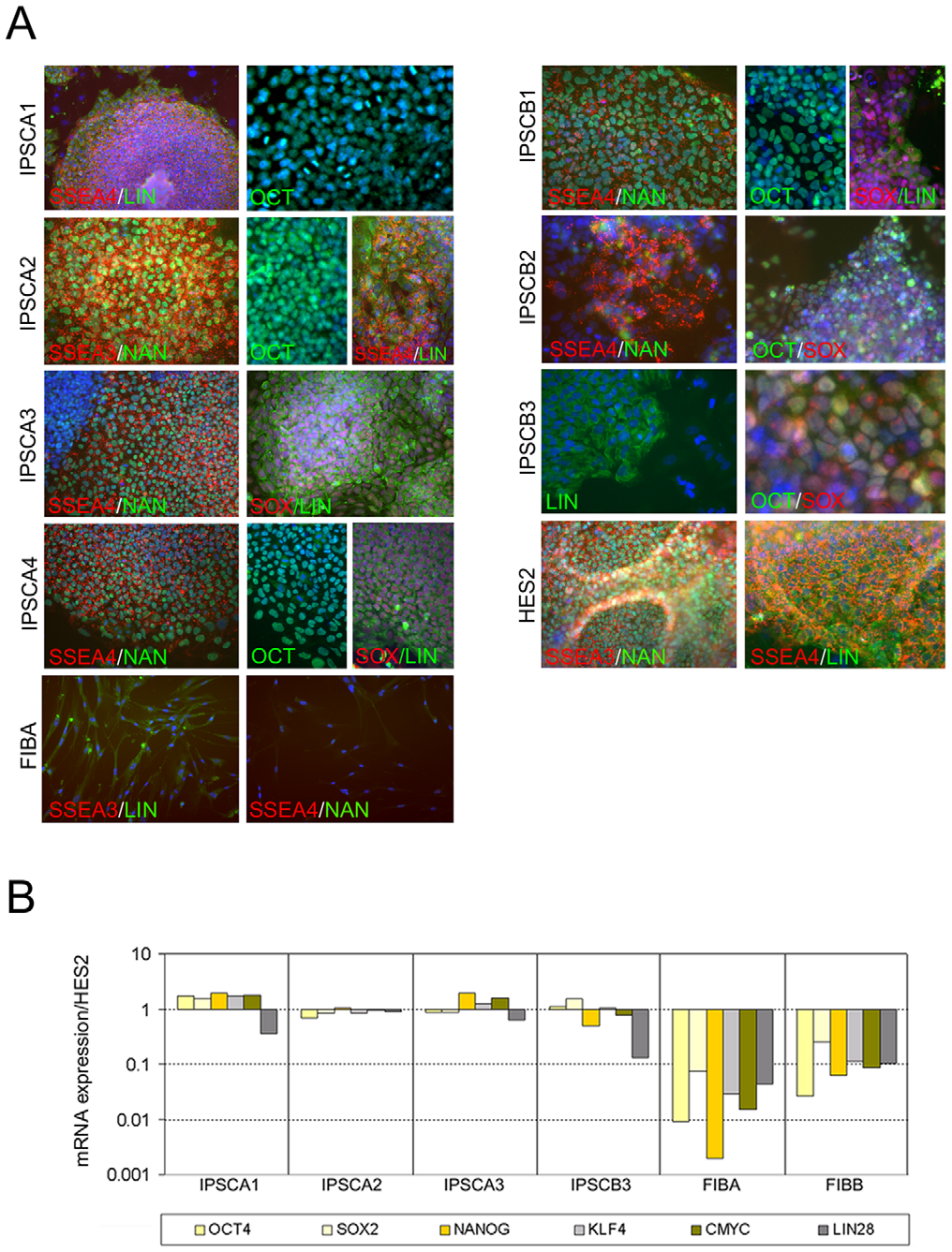
Indicators of cell phenotype and pluripotency in IPSC lines. (A) Immunofluorescent analysis of pluripotency markers as labeled. Label color corresponds to marker color in images. Blue staining in all panels is DAPI labeling of nuclei. All IPSC lines displayed strong positive expression of pluripotency markers that were not observed at high levels in input FIB cells with the exception of the Lin28 antibody that reproducibly produced faint fluorescence in the cytoplasm of fibroblasts. (B) Representative QPCR analysis of FIB lines and reprogrammed IPSCs for markers of pluripotency and reprogramming factors relative to factor levels in hESCs. Input fibroblasts express very low levels of most factors whereas IPSCs display levels very similar to hESCs.

To determine if human somatic cells undergo telomere elongation when reprogrammed to pluripotency, primary fibroblasts from human subjects of two age extremes – 16 weeks of gestation (line FIBA), and 70-years of age (line FIBB) – were converted to IPSCs by expression of reprogramming factor combinations described by Yu et al. (2007)[14] and Takahashi et al. (2007)[15] using lentiviral vectors shown in Figure 1. IPSC and other cell lines used in this report are summarized in Table 1. IPSCs derived from each fibroblast line displayed the characteristic colonies of tightly packed round cells with relatively large nucleus/cytoplasm ratios and prominent nucleoli characteristic of hESCs (Figure 2). Immunochemical and qPCR analysis of reprogramming factors in IPSCs from both donors revealed that they differed dramatically from input fibroblasts in marker expression, but displayed immunostaining patterns (Figure 3A) and expression levels of reprogramming factors (Figure 3B) that were very similar to hESCs. Microarray analysis of several IPSC lines compared to input fibroblasts and hESCs further confirmed their similarity to hESCs and loss of fibroblast identity (data not shown).

**Table 1:** Cell lines used in this report.

Report Name	Cell Type	Pass	Parental Line	Vec/Factors	Line Ref Name
IPSCA1	IPSC	5–6	FIBA	OSNL	MSUH-001
IPSCA2	IPSC	5–6	FIBA	OSNL	MSUH-002
IPSCA3	IPSC	5–6	FIBA	(ON)(SL)	MSUH-004
IPSCA4	IPSC	5–6	FIBA	OSNL	MSUH-005
IPSCB1*	IPSC	5–6	FIBB	OSKM	MSUH-006*
IPSCB2*	IPSC	5–6	FIBB	OSKM	MSUH-007*
IPSCB3	IPSC	5–6	FIBB	OSKM	MSUH-008
FIBA	FIBROBLAST	15–16#	NA	NA	IMR90
FIBB	FIBROBLAST	5–6#	NA	NA	MSUH-004F2
TERA2	TERAT-DERIVED	4–5	IPSCA2	OSNL	MSUH-002T1
TERA3	TERAT-DERIVED	4–5	IPSCA3	(ON)(SL)	MSUH-003T1
TERB3	TERAT-DERIVED	4–5	IPSCB3	OSKM	MSUH-008T1
HES1	ES CELL	80	EMBRYO	NA	H7
HES2	ES CELL	44	EMBRYO	NA	H9

*Report name*: name used to reference the cell lines in this report. *Cell Type*: the general cell phenotype.

*Pass:* passage of cells at time of telomere analysis. *Parental Line*: name of the input cell line used to produce the corresponding cell line.

*Vec/Factors*: Reprogramming factor combination used to produce cell line (if applicable). Individual letters represent each of the 6 reprogramming factors as in Figure 1. Parentheses indicate coupling of factors into bicistronic pairs in the vector.

*Line Ref Name:* Official name of each cell line used in the report.

* Lines lost to contamination.

# --also passage number used for production of IPSCs.

**Figure 4:**
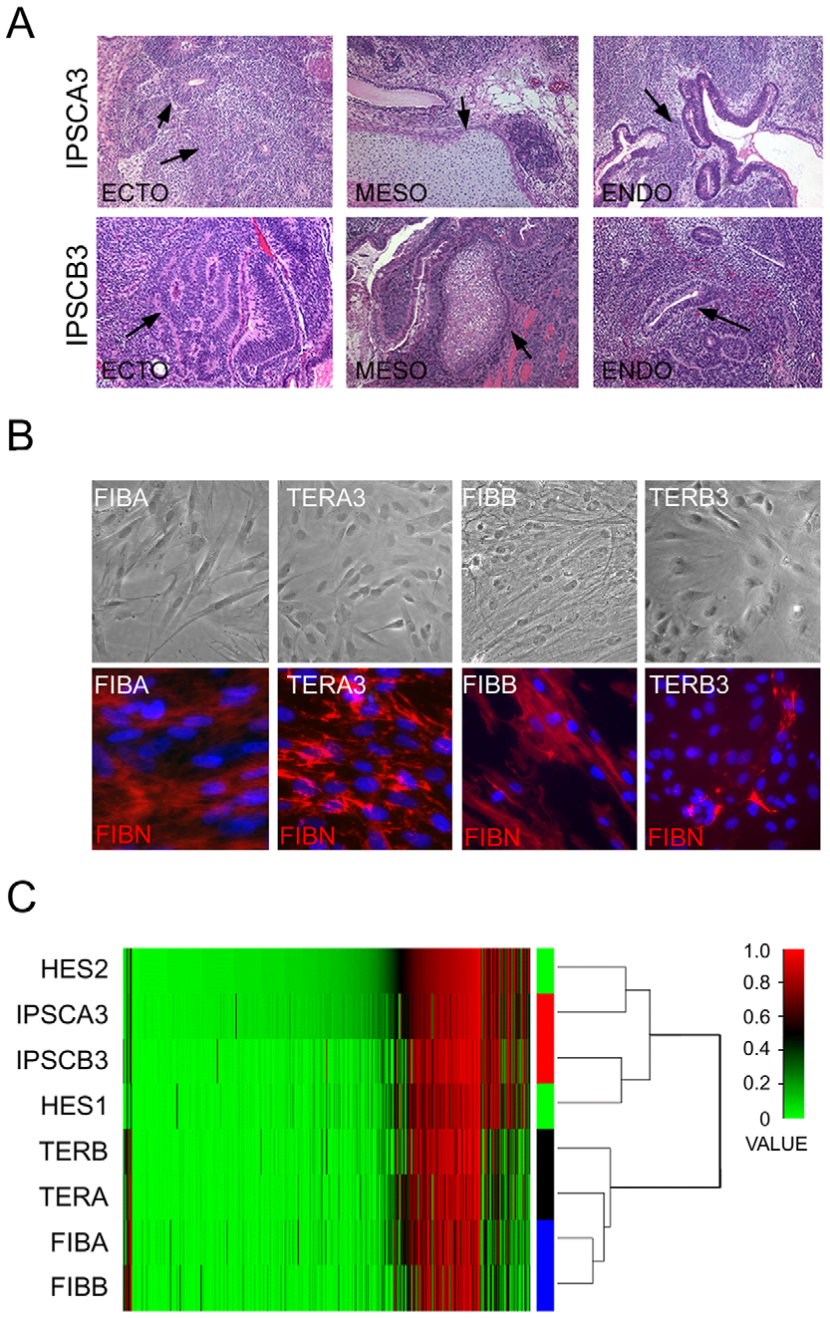
Indicators of pluripotency and differentiation in IPSC lines. (A) Representative sections from teratomas generated from IPSC lines as labeled. Arrows in ectodermal (ECTO) tissue indicate neural rosettes, in mesodermal tissue (MESO) indicate cartilage, and in endodermal tissue (ENDO) indicate glandular columnar epithelium. mag. 100X. (B) Morphology of FIB lines and TER lines, as labeled, in phase-contrast images (top row) and stained for fibronectin (red, bottom row). mag. 400X. Blue is DNA stain. (C) Genome-wide methylation heat map and cluster analysis of representative input fibroblasts, IPSCs, TER, and hESC lines indicating that the overall pattern of methylation of IPSCs closely matches HES lines while differentiated TER lines cluster with input fibroblasts. Value indicates the level of methylation ranked from 0 (hypomethylated) to 1 (hypermethylated).

FIBA- and FIBB-derived IPSCs gave rise to teratomas with tissues representing all three germ layers indicating that they are pluripotent (Table 1 and Figure 4A). IPSCs were returned to a differentiated phenotype by dissociating teratoma tissue *in vitro* and culturing and passaging outgrowing cells under conditions that favored the expansion of cells with fibroblast characteristics. These teratoma-derived cells (TER cells) displayed a morphology similar to input fibroblasts and many cells were immunopositive for fibroblasts markers such as fibronectin (Figure 4B). DNA methylation analysis of FIB lines, IPSCs, and TER cells revealed that TER cells clearly clustered with input fibroblasts (r = 0.88) while IPSCs clustered with human ESCs (r = 0.97) (Figure 4C). Together, these data established the identity of the IPSC lines as pluripotent cells and TER lines as differentiated cells sharing many qualities of input FIB lines.

### Telomere Elongation after IPSC Conversion and Telomere Shortening after IPSC Differentiation

**Figure 5:**
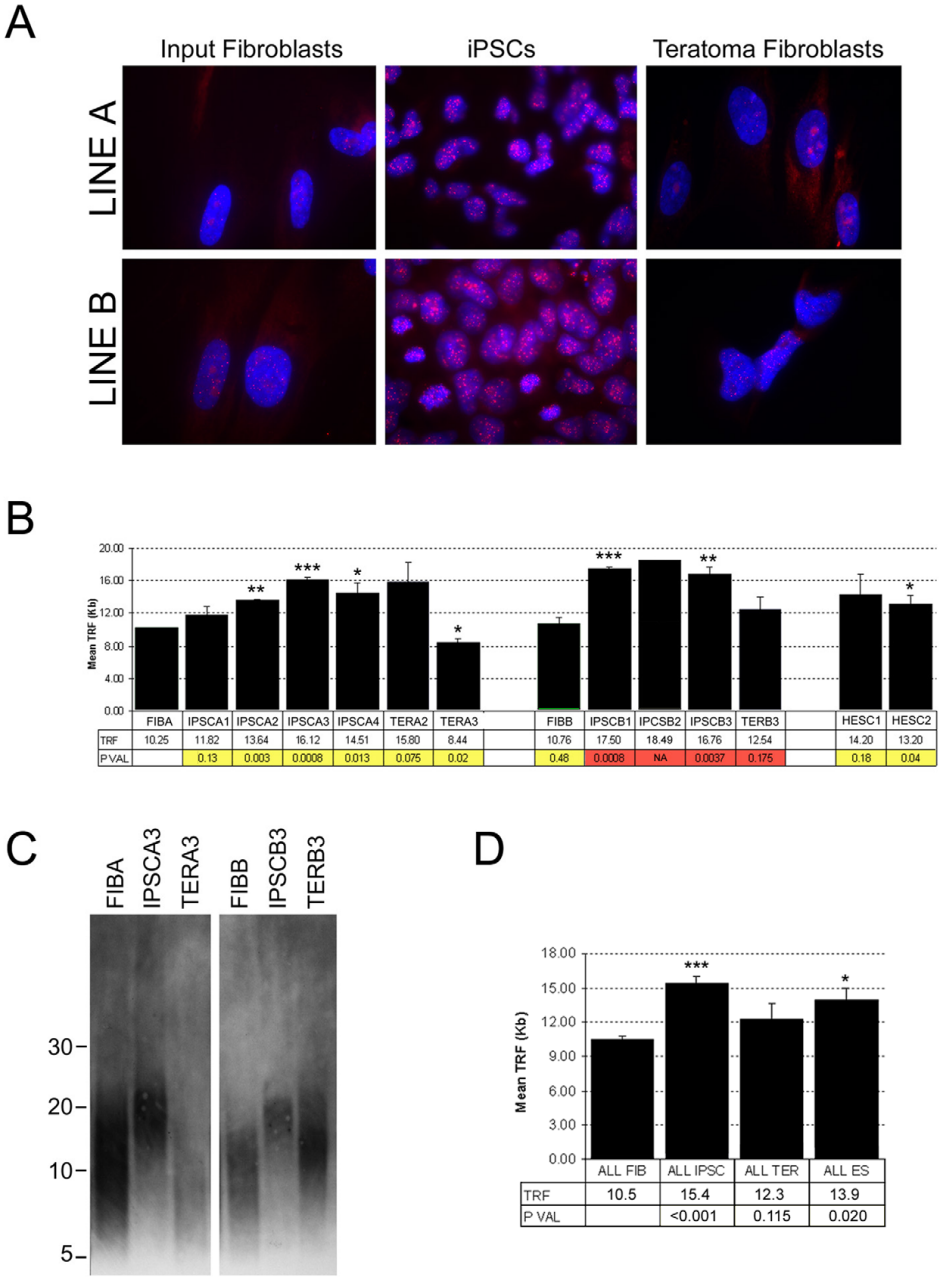
Analysis of telomeres in input, pluripotent, and re-differentiated cell lines. (A) Blue-stained nuclear DNA with punctate red fluorescence indicating telomeres hybridized to the PNA FISH telomere probe for input fibroblasts, IPSCs, or TER cells from lines A and B as labeled. mag. 400X. (B) Quantification of TRFs from cell lines as labeled. Yellow shading indicates significance compared to line FIBA and red shading significance compared to line FIBB. TRF indicates mean length in kilobase pairs (Kb). * = P<0.05, ** = P<0.01, *** = P<.001. Error bars indicate SEM. (C) Example of changes in TRF size in cell lines as labeled. Numbers at left are fragment lengths in kilobases. (D) Mean TRF lengths for all cell lines combined by type. Labeling as in part B. Significance calculated relative to FIB.

Initial analysis of telomeres was performed by fluorescent *in situ* hybridization (FISH) [19], [20] using a telomere peptide nucleic acid (PNA) probe on cultures of each class of cell at P5–6. Samples processed in parallel clearly revealed intensified punctate fluorescence characteristic of labeled telomeres in the IPSCs of both line A and line B relative to either input cells or TER cells (Figure 5A). Intensified fluorescence in the IPSC cultures relative to input cells and redifferentiated TER cells suggested that telomeres in pluripotent IPSCs were significantly elongated compared to the differentiated cell types.

Telomeres in FIB, IPSC, and TER cells were further analyzed and quantified using TRF analysis [9], [21], a method based on Southern blot hybridization of restriction endonuclease digested genomic DNA with a labeled telomere-repeat probe. Fibroblast lines A and B, seven IPSC lines, three TER lines, and two human ESC lines were examined and quantified as shown in Figure 5B–D. FIBA displayed a mean TRF length of approximately 10 Kb and three IPSC lines derived from FIBA were found to have significantly (P<0.05) elongated telomeres of between 13.6 and 16.1 Kb (Figure 5B). Line IPSCA1 also displayed indications of elongation, but did not reach statistical significance. Line FIBB displayed a telomere length that did not differ statistically from FIBA (10.7 Kb), and telomere elongation was observed in all IPSC lines derived from FIBB (Figure 5B) (because there was only sufficient DNA for a single run of IPSCB2, statistical significance could not be calculated for this sample).

In IPSCs differentiated into TER lines, TERA3 and TERB3 displayed a significant loss of telomere length relative to the parental IPSC line (P<0.001 for TERA3 and P = 0.04 for TERB3) and TERA3 even showed a slight but significant decrease compared to FIBA (P = 0.02). Notably, however, the TERA2 line displayed elongated telomeres relative to both the parental fibroblast and IPSC lines. This difference was not statistically significant due to variation in TRF lengths in different samples of TERA2 even at approximately the same passage, suggesting that the TERA2 is not homogenous and likely contains a subpopulation of cells that are continuing to elongate telomeres and may be more rapidly proliferating. Comparing all lines, we found that an average increase of approximately 47% (from 10.5 Kb to 15.4 Kb) accompanies conversion to the pluripotent phenotype, and following differentiation, telomere length decreases to levels approximating input fibroblasts (Figure 5D).

## Discussion

Though the IPSC lines in this report were isolated from only two subjects of very disparate ages, these results suggest that like animal cells reprogrammed to pluripotency, reprogrammed human somatic cells will generally restore telomeres to lengths characteristic of human ESCs. It follows that IPSC-derived cells will not reach replicative senescence prematurely, regardless of donor subject age.

Similar to what was observed in mouse IPSCs, our results also suggest that variables in the IPSC conversion process including the age and sex of input cells, reprogramming factors used (Oct4 and Sox2 coupled with either Nanog and Lin28 or KLF4 and c-Myc), and media conditions for early establishment (HES medium with either 100 ng/zFGF or 4 ng hFGF) do not preclude telomere elongation in human IPSCs nor shortening following redifferentiation.

In general, by P5, human IPSCs displayed TRFs that equaled early passage hESCs. This differs from mouse IPSCs that, as pointed out in the Introduction, did not attain mESC telomere lengths until P30 [9]. Whether human IPSCs undergo a progressive increase over a much shorter time frame (such as between passage 1–5), or are essentially “born” with elongated telomeres accompanying conversion to the pluripotent phenotype, has not been determined; however, given that P5 approximates the earliest point at which human IPSC lines for either the generation of more differentiated cell types or therapeutic application can reasonably be used, an answer to this question has little practical relevance. Furthermore, it is possible that the difference in telomere elongation in mouse IPSCs relative to human IPSCs is a simple function of average telomere length. It has been long-established that mouse chromosomes bear telomeres an order of magnitude longer than their human counterparts [22], and perhaps the elongation process in mouse takes more cell cycles simply because the telomeres are much longer. In either event, the lengths of telomeres in both the human IPSCs and hESCs described in this report fall well within the range of human ESC telomeres described previously [11], [13]

One aspect of telomere dynamics that had not been studied for IPSCs of any species prior to this report is resumption of telomere shortening following redifferentiation of the cells. While both ESCs and IPSCs can be differentiated *in vitro* by cell culture methods with some success, we chose to use cells cultured from teratomas because prior to plating, these cells undergo many weeks of growth and replication in an environment conducive to differentiation. Although cells of a variety of differentiated phenotypes were observed in primary cultures from IPSC teratomas (data not shown), repeated passage of the most adherent population in fibroblast medium rapidly led to a relatively homogenous population of cells with a flattened morphology reminiscent of fibroblasts.

Although telomeres were reduced in two of the TER lines isolated, in a third – TERA2 – elongated telomeres were maintained. Given that only one line acted contrary to expectation and retained elongated telomeres following extended growth *in vivo* and additional culture *in vitro*, we can only speculate as to the relevance of this observation. The two most likely explanations are that either a subpopulation of IPSCs have retained their pluripotent cell identity despite extended growth and differentiation in conditions that do not support IPSC/ESC maintenance, or a subpopulation of cells have taken on a non-pluripotent cell phenotype that supports telomere elongation, such as a transformed cell [23]–[25]. Analyses of the TERA2 line for indicators of a pluripotent cell population have thus far proved negative (data not shown). While the observation that the TERA2 cell population retains elongated telomeresthe ovser may raise cautionary flags regarding the use of uncharacterized IPSCs *in vivo*, in general, the results with TER lines indicate the results with the that most IPSCs will resume normal telomere physiology after differentiation. Nevertheless, even if the emergence of cells with an extended capacity for self-renewal from differentiated IPSC populations is a rare event, this finding suggests that it may be prudent to examine the telomere dynamics of individual IPSC lines destined for extensive use in either cell culture models or future therapeutic applications.

## Materials and Methods

### Cell Culture, IPSC Production, and EB/Teratoma

Fibroblasts and TER cell lines were cultured in DMEM+10%FBS+antimycotic/antibiotic (FIB medium). FIBA (IMR90) was obtained from ATCC and FIBB was grown out from a skin biopsy from a 70-year old male subject with informed consent. Human ES cell line HES2 (H9) was obtained the University of Wisconsin and was used between passages 43–44 for analyses in this report. DNA from HES1 cells (H7) at approximately P80 was obtained from Sue O'Shea's laboratory at the University of Michigan.

IPSCs were produced from FIBA cells at P15 and FIBB cells at P5. IPSCs were generated by infection with high-titer lentiviral vectors encoding the six human reprogramming factors Oct4 (O), Sox2 (S), KLF4 (K), cMyc (M), Nanog (N), and Lin28 (L) expressed singly essentially as described [14], [15] or in bi-cistronic pairs (see Figure 1). The lentiviral vector used was either the SIN-EF1a vector described by [14] and obtained from Addgene, a lentiviral vector bearing a PGK promoter for Sox2 as shown in Figure 1 (Sox2 expression appeared more robust from the PGK vector). Vectors were modified to encode KLF4, cMyc (Obtained from Open biosystems) or reprogramming factors were co-expressed using 2A elements as shown in Figure 1. Infection, identification of IPSCs, subcloning of IPSC colonies, and passage onto MEFs, was performed essentially as described in [14] for OSLN lines and as described in [15] for OSKM lines. After initial derivation, all lines were maintained as in [14].

TER lines were isolated from explanted teratomas (generated from P5 IPSCs) by removing the tissue from sacrificed teratoma-bearing mice and washing the tumor tissue extensively in sterile PBS containing 10X antibiotic/antimycotic. Tissue not central to the teratoma was removed using forceps and a scalpel, and the remaining tissue minced and distributed in a 10-cm plate with minimal (5 ml) FIB medium overnight to allow attachment. After 7–10 days of culture with regular medium changes, large explant pieces were removed by suction and the remaining outgrowth passaged. After preplating for 1–2 hours, loose cells and cell debris was removed and the medium replaced. By passaging every 4–6 days for 1 month, TER lines ultimately displayed a uniform fibroblastic-like appearance by passage 5–6 when they were harvested for DNA and other analyses.

Embryoid bodies were made using standard methods that include transferal of manually detached IPSC colonies onto low-adherence plates accompanied by differentiation in 20% FBS medium. After two weeks, EBs were collected, transferred to tissue culture plates where they re-adhered, and ultimately passaged in fibroblast medium. Teratomas were produced in nude mice using standard methods. Briefly, 1×10^6^ IPSCs of each line at P5 were suspended in DMEM/F12, and injected into Nude mice intramuscularly. 6–8 weeks after injection, tumors were surgically dissected and either processed as described above to isolate TER lines, or fixed in 4% paraformaldehyde, embedded in paraffin, sectioned, and processed for histological examination. Tissue processing was performed by MSU histology core facility.

### Analysis of Cell Phenotypes

IPSC lines were cultured under standard conditions to passage 3–4 for harvest to produce RNA for QPCR analysis, perform immunocytochemistry, or other analyses. FIB and TER lines were analyzed at the passages listed in Table 1. Fibronectin monoclonal antibody HFN7.1, SSEA3 and SSEA4 antibodies were obtained from the Developmental Studies Hybridoma Bank at the University of Iowa and Oct4, Nanog, and Lin28 antibodies were obtained from Santa Cruz Biotechnology (Santa Cruz, CA). All primary antibodies were used at 1∶250. Secondary anti-mouse, anti-goat, and anti-rabbit-Alexa conjugates were obtained from Invitrogen and used at 1∶1000. RNAs were isolated using Trizol reagent (Invitrogen) following the manufacturers protocol. Immunochemical analyses used to confirm cell phenotypes were performed on cultured cells fixed with 4% paraformaldehyde and processed using standard methods. Pluripotent cells used for methylation analysis and for TRF analysis (below) were harvested by manual microdissection of colonies with good ESC-like morphology to remove cells at the edges that were partially differentiated and most MEFs from the feeder layer. The genome-wide methylation signature of bisulfite-converted DNA from all cell lines was performed by the Applied Genomics Technology Center, Wayne State University, using the Illumina HumanMethylation27 BeadChip.

### FISH and TRF Analysis

For FISH analysis, cells at the passages listed in Table 1 were washed with PBS and fixed with 4% paraformaldehyde for 10 minutes at room temperature. After washing, cells were treated with 0.005% Pepsin (Sigma) for 10 minutes at 37°C and immediately dehydrated with ice-cold ethanol. Fluorescent telomere PNA probe (Panagene) was diluted in 10 mM Tris-HCl containing 70% formamide to a final concentration of 180 nM. Samples were incubated with the probe for 2 hours at room temperature and were visualized with an epifluorescence system on a Nikon TE-2000 microscope.

Analysis of telomere restriction fragment lengths [9], [23] was performed using TeloTAGGG (Roche) reagents and following the manufacturers protocol. !-2ug of DNA from cells at the passages listed in Table 1 were digested overnight with RsaI/HinfI, run the next day on a 0.8% agarose gel, and transferred to nylon membrane for 48 hours for Southern analysis. Cell lines were harvested for DNA on at least two separate occasions (except for HES1 which was harvested only once) and run 2–6 times to determine mean TRF length. There was only sufficient DNA for a single run of IPSCB2 due to loss of this line to contamination. Images were analyzed using NIHImageJ and TRF lengths were calculated relative to labeled molecular weight markers. Statistical significance was analyzed using Student's T-test

### Ethics Statement

This study was conducted according to the principles expressed in the Declaration of Helsinki. The study was approved by the Institutional Review Board of Michigan State University, reference: #06-699 for the study titled: “Direct Dedifferentiation of primary somatic cells”. All patients provided written informed consent for the collection of samples and subsequent analysis.
