# Correction: Telomere Dynamics in Human Cells Reprogrammed to Pluripotency

**DOI:** 10.1371/annotation/c786d141-fd0f-45fd-80ec-96d80be620dc

**Published:** 2010-03-19

**Authors:** Steven T. Suhr, Eun Ah Chang, Ramon M. Rodriguez, Kai Wang, Pablo J. Ross, Zeki Beyhan, Shashanka Murthy, Jose B. Cibelli

The first two authors, Steven T. Suhr and Eun Ah Chang, should be noted as having contributed equally to this work. 

